# Neuroimmune interplay during type 2 inflammation: Symptoms, mechanisms, and therapeutic targets in atopic diseases

**DOI:** 10.1016/j.jaci.2023.08.017

**Published:** 2023-08-25

**Authors:** Brian Kim, Marc E. Rothenberg, Xin Sun, Claus Bachert, David Artis, Raza Zaheer, Yamo Deniz, Paul Rowe, Sonya Cyr

**Affiliations:** a Kimberly and Eric J. Waldman Department of Dermatology, Mark Lebwohl Center for Neuroinflammation and Sensation, Icahn School of Medicine at Mount Sinai, New York; b Division of Allergy and Immunology, Department of Pediatrics, Cincinnati Children’s Hospital Medical Center and University of Cincinnati College of Medicine; c Department of Pediatrics, University of California, San Diego; d Department of Otorhinolaryngology, Head and Neck Surgery, University of Muenster; e First Affiliated Hospital, Sun Yat-Sen University, International Airway Research Center, Guangzhou; f Jill Roberts Institute for Research in Inflammatory Bowel Disease, Friedman Center for Nutrition and Inflammation, Joan and Sanford I. Weill Department of Medicine, Department of Microbiology and Immunology, Weill Cornell Medicine, Cornell University, New York; g Sanofi, Cambridge; h Regeneron Pharmaceuticals Inc, Tarrytown; i Sanofi-Aventis, Bridgewater.

**Keywords:** Type 2 inflammation, sensory neurons, cytokines, neuropeptides, atopic dermatitis, prurigo nodularis, asthma, chronic rhinosinusitis with nasal polyposis, eosinophilic esophagitis, neuroimmune

## Abstract

Type 2 inflammation is characterized by overexpression and heightened activity of type 2 cytokines, mediators, and cells that drive neuroimmune activation and sensitization to previously subthreshold stimuli. The consequences of altered neuroimmune activity differ by tissue type and disease; they include skin inflammation, sensitization to pruritogens, and itch amplification in atopic dermatitis and prurigo nodularis; airway inflammation and/or hyperresponsiveness, loss of expiratory volume, airflow obstruction and increased mucus production in asthma; loss of sense of smell in chronic rhinosinusitis with nasal polyps; and dysphagia in eosinophilic esophagitis. We describe the neuroimmune interactions that underlie the various sensory and autonomic pathologies in type 2 inflammatory diseases and present recent advances in targeted treatment approaches to reduce type 2 inflammation and its associated symptoms in these diseases. Further research is needed to better understand the neuroimmune mechanisms that underlie chronic, sustained inflammation and its related sensory pathologies in diseases associated with type 2 inflammation.

Type 2 immunity is a specialized, evolutionarily conserved arm of the immune system that combats ectoparasitic and endoparasitic helminths, expels toxins, and promotes tissue repair.^1–4^ When the epithelial barrier is breached, alarmin cytokines (eg, thymic stromal lymphopoietin [TSLP], IL-25, IL-33) activate tissue-resident immune cells (such as mast cells, dendritic cells, and group 2 innate lymphoid cells [ILC2s]) while simultaneously recruiting granulocytes, including eosinophils and basophils. Collectively, these cells orchestrate a polarized type 2 immune response through type 2 cytokines, histamine, and other mediators that neutralize and expel parasitic helminths and toxins and repair the barrier through epithelial turnover, remodeling, and fibrosis. Although these processes are protective and intended to restore tissue homeostasis, in the setting of allergy and continuous barrier stress they become pathologic, resulting in a variety of chronic inflammatory diseases.

Mechanical reflexes such as scratching, airway constriction, coughing, sneezing, and gastrointestinal motility also protect barrier surfaces and are triggered by direct activation of sensory neurons, often in concert with autonomic input to the target organs. Many of these manifestations are pathologically altered in diseases with type 2 immune dysregulation (Fig 1), including (but not limited to) atopic dermatitis (AD), prurigo nodularis (PN), asthma, food allergy, chronic rhinosinusitis with nasal polyps (CRSwNP), and eosinophilic esophagitis (EoE).^1,2,5,6^ The extent of similarities among the neuroimmune pathways regulating these mechanical responses remains to be fully defined (Table I^7–73^).

Type 2 inflammation is characterized by overexpression and increased activity of type 2–associated cytokines (including IL-4, IL-5, IL-13, and IL-31) and alarmins (including IL-25, IL-33, and TSLP). An important property of these cytokines is their ability to activate or sensitize peripheral sensory neurons. For example, IL-4, IL-13, and IL-31 trigger rapid calcium responses in dorsal root ganglion (DRG) neurons and promote itch,^18,19,74^ and IL-5 has been implicated in the release of vasoactive intestinal peptide (VIP) from sensory neurons in the lungs.^75^ At the same time, sensory neurons produce neuropeptides such as VIP, substance P (SP), and calcitonin gene–related peptide (CGRP), which act directly on vascular endothelial cells and smooth muscle cells to mediate vascular effects, as well as on immune cells (Fig 2).^61,76–80^ Neurons and immune cells colocalize in affected areas of the skin in AD and PN and in the airways in asthma.^7,8,53^ In addition, patients with AD and PN often show alterations in nerve fibers in affected tissue.^10,42,43,81^ IL-31, a type 2 cytokine associated with itch, has been shown to promote sensory nerve elongation, branching, and density both *in vitro* and in lesional skin in mice.^82^ In asthma, sensory neurons also exhibit hyperinnervation; for example, IL-5 exposure *in utero* enhances nerve sprouting in lungs in mice.^83^

The mechanisms by which sensory neurons, their associated neuropeptides, and tissue-resident or infiltrated immune cells interact to activate or regulate specific pathways of inflammation, and how these histologic alterations affect the precise sensory response and neuroinflammatory function of organs across diseases featuring type 2 immune dysregulation (including AD, PN, asthma, CRSwNP, and EoE) remains a major area of inquiry. Herein, we review current research on these pathways and present recent advances in targeted treatments to reduce type 2 inflammation and associated sensory pathologies in these diseases.

## NEUROIMMUNE CROSS TALK AND SENSORY PATHOLOGY IN CONDITIONS ASSOCIATED WITH TYPE 2 INFLAMMATION

### The itch-scratch cycle in AD and PN

AD and PN are chronic inflammatory skin disorders featuring intense pruritus and an itch-scratch cycle.^84^ Both diseases are often accompanied by pruritic skin, sleep disturbances, and impaired quality of life.^44,85^ However, there are notable differences. AD is a relapsing-remitting type 2 inflammatory disease characterized by red, scaly, oozing, and crusted lesions, as well as by increased risk for cutaneous infections and intermittent flares.^86–90^ In contrast, PN is a chronic pruritic condition with type 2 immune dysregulation with or without atopy. In addition to intense pruritus lasting for 6 weeks or longer, PN features discrete papulonodular lesions with firm fibrotic qualities.^85,91,92^ Although AD is considered a primary inflammatory dermatosis that secondarily results in pruritus, PN may be initiated by sensory neuron dysfunction, resulting in itch that is triggered independently of a pruritogen stimulating the neuron that can cause secondary fibrotic papulonodular lesions as a result of mechanical disruption of the skin from vigorous scratching.^44^

**FIG 3.**
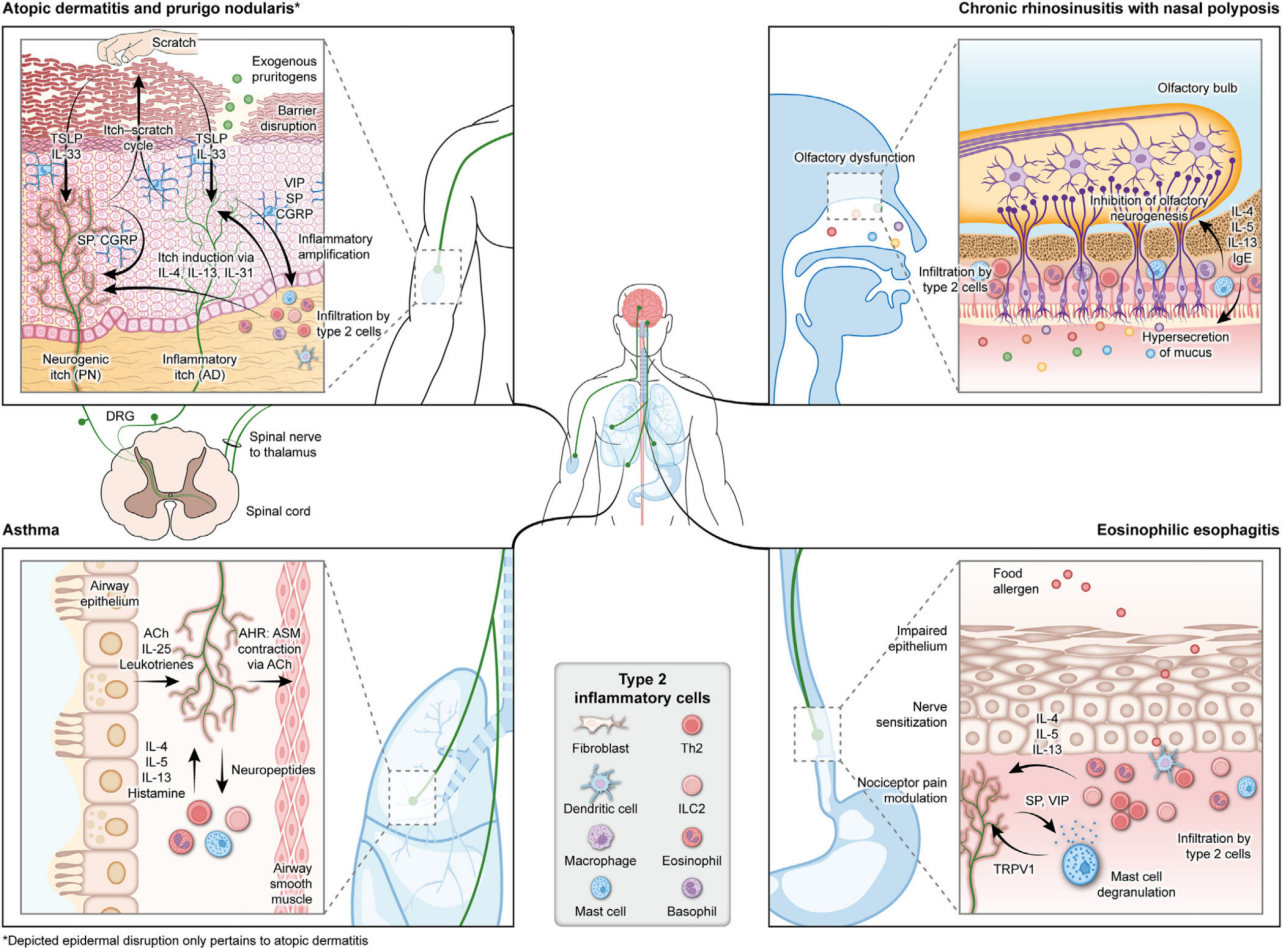
Neuroimmune mechanisms underlying sensory pathologies in conditions associated with type 2 inflammation. *Depicted epidermal disruption pertains only to AD. *ACh*, Acetylcholine; *ASM*, airway smooth muscle.

The itch-scratch cycle refers to a vicious cycle in which pruritus drives repeated scratching, which in turn further exacerbates pruritus either directly by mechanical sensitization of nerves or indirectly via release of trauma-induced inflammatory mediators. In this cycle, abnormal neuroimmune cross talk promotes a positive feedback loop that drives sensitization to pruritogens and itch amplification (Fig 3).^11^

Multiple and overlapping mechanisms can lead to itch, which can be acute or chronic. Triggers include exogenous pruritogens, endogenous pruritogens produced by keratinocytes and immune cells, and mechanical stimuli (eg, from light touch).^93,94^ In a genome-wide association study, exogenous itch from mosquito bites showed a strong association with cytokine pathway loci, including interferon regulatory factor 1 (*IRF1*), *IL3*, colonystimulating factor 2 (*CSF2*), and *IL21*, along with a weaker association with other loci, including IFN-γ–AS1 (*IFNG-AS1)* and signal transducer and activator of transcription (STAT) 6 (STAT6).^95^ In addition, acute itch is initiated primarily via histamine release from mast cells and basophils through degranulation following activation by allergens via IgE or via inflammatory mechanisms, as seen in acute urticaria and, less commonly, in chronic urticaria.^48^ Mechanical stimuli activate Piezo1 channels on itch sensory neurons. In mouse models, stimulation of Piezo1 evoked alloknesis (defined as an itch response evoked by light touching of skin, particularly in dry or aging skin).^94^ This suggests that itch-specific sensory neurons, which also express IL-4 receptor alpha (IL-4Rα),^18^ could be pathologically stimulated via type 2 neurogenic inflammation, and itch could be further amplified by excessive touch or scratching via the Piezo1 transducer. Nonhistaminergic mediators of acute itch have also been identified (leukotriene C_4_, oncostatin M [OSM], bovine adrenal medulla peptide 8–22 [BAM8–22], and SLIGRL-NH_2_).^96–102^ Additionally, many of these mediators are variably dependent on canonic transient receptor potential ankyrin 1 (TRPA1) and transient receptor potential vanilloid 1 (TRPV1).^99–102^ Further, a number of itch-mediating G protein–coupled receptors (GPCRs) have been identified in both mice and humans; they include the *Mas*-related family of GPCRs (eg, *Mas*-related family of G-protein-coupled receptor A3 [MrgprA3] and MrgprC11 in mice and *Mas*-related family of G protein-coupled receptor X1 [MRGPRX1] in humans).^96–103^

The IL-4– and IL-13–mediated Janus kinase (JAK) and STAT intracellular signaling pathways are critical for T_H_2 cell differentiation and type 2 inflammatory processes. Inhibition of JAK/STAT signaling can improve clinical symptoms of AD, including itch.^37,41,104^ Binding of IL-4 and IL-13 to IL-4Rα activates JAK1, JAK3, tyrosine kinase 2, and STAT6. These molecules play an important role in triggering type 2 inflammatory responses, including pruritic responses, as well as acute pruritic responses to stimuli such as insect bites.^38,95,105–107^ Importantly, STAT6 gain of function is associated with chronic atopic conditions in humans.^106,108–110^ Although exogenous itch such as that from insect bites has been associated with type 1 and type 2 cytokines, evidence suggests that chronic inflammatory itch is associated with the IL-4/IL-13/JAK1/STAT6 axis.^95,105–107^

Chronic itch in AD and PN is induced largely via non-histaminergic mediators. Type 2 inflammatory cytokines activate nonhistaminergic itch in multiple ways.^11^ In response to pruritic stimuli, scratching-induced skin stress or damage (eg, resulting from dry skin) can cause keratinocytes to release alarmins, including TSLP and IL-33, which can trigger pruritus by acting directly on sensory neurons.^11,14–16^ IL-33 may also play an indirect role in severe itch by initiating the IL-33/ST2/CGRP axis, which has been shown to promote interactions between type 2 inflammatory cells and somatosensory neurons to induce itch in allergic conjunctivitis.^111^ Notably, a clinical trial of the anti-TSLP mAb tezepelumab in patients with moderate-to-severe AD found little to no efficacy in itch (versus that with placebo), suggesting that TSLP may have only a limited role in pruritus in AD.^17^ Type 2 cytokines and related factors (such as OSM) may have a prominent role in chronic pruritus and sensitization to pruritogens. Both IL-4 and IL-31 are implicated in the transmission of the pruritic sensation in atopic diseases, with IL-4 promoting T_H_2 cell polarization, thereby contributing to increased IL-31 expression levels observed in both AD and PN lesional skin.^9^ When compared with their expression in samples from nonatopic patients, expression of IL-4 and IL-13 (both of which bind to IL-4Rα) is increased in patients with AD lesions.^112^ A subset of neurons coexpress neuropeptides and receptors for type 2 cytokines, including IL-4Rα and IL-31 receptor A (IL-31RA)^18^; for example, some sensory neurons in skin coexpress IL-31RA, TRPV1, and TRPA1 in the DRGs.^19^ These cation channels mediate the release of neuropeptides such as CGRP and SP and are essential for the itch-inducing effects of IL-31, thus providing a direct link between type 2 inflammatory cytokines and neurogenic inflammation.^19^ IL-4Rα stimulation activates sensory neurons and enhances their response to pruritogens (such as IL-31 and histamine) via transient receptor potential channels and JAK1 pathways.^11,18,84,97^ In human sensory neurons, exposure to type 2 cytokines (eg, IL-4, IL-13, IL-25, IL-31, IL-33, TSLP) increases sensory neuron excitability, amplifies the neuronal response to pruritogens, and alters activity of downstream gene transcription factors related to AD and itch (including TRPV1 and TPRA1).^18,113,114^ OSM, which shares a coreceptor with IL-31, also promotes sensitization of itch-specific neurons and is correlated with itch intensity.^49,97^

The release of neuropeptides from sensory neurons in the skin in response to mechanical or chemical stimuli produces multiple proinflammatory effects, including release of proinflammatory cytokines and neuropeptides from skin cells, and activation of a variety of immune cells.^10–12,48^ Mast cells are particularly well positioned to act as a link between the nervous and immune systems, as they are often found near afferent nerve fibers.^7,8^ Many neuropeptides have direct effects on mast cells, promoting mast cell degranulation and the production of type 2 cytokines and chemokines.^7^ Peptides, including SP, proadrenomedullin peptide, VIP, CGRP, and pituitary adenylate cyclase-activating polypeptide, have been implicated as ligands for MRGPRX2, a key receptor that mediates neuropeptide activation of mast cells.^115–118^ Basophils can also interact with sensory neurons, mediating allergen-induced itch in the context of AD-associated inflammation.^13^ Interestingly, basophils have been reported to express MRGPRX2, demonstrating the diversity of ways in which granulocytes engage in a bidirectional positive neuroimmune feedback loop that may facilitate and maintain states of pathologic sensation and chronic inflammation.^119^

In AD, plasma SP concentrations are linked to pruritus intensity, and in PN, lesional skin demonstrates an elevated density of SP- and CGRP-immunoreactive nerve fibers.^42,43,45–47^ VIP concentrations are also higher in patients with AD than in individuals without AD.^120^ Proteases derived from house dust mites are common allergens that trigger the release of SP from TRPV1-expressing sensory neurons, which activates mast cells via MrgprB2 in mice and recruits dendritic cells that prime type 2 differentiation.^61,80^ In addition, scratching may stimulate the release of neuropeptides, including CGRP, nerve growth factor, and VIP.^11,84^ Taken together, these findings suggest that allergens and other environmental stimuli may directly stimulate both itch and neurogenic inflammation via neuropeptides such as SP.

The extracellular matrix protein periostin is overexpressed in the skin of patients with AD and may also contribute to itch. TSLP induces periostin secretion in keratinocytes, which promotes JAK/STAT signaling and T_H_2 cell immunity; it also interacts with integrins to promote immune cell activity and directly stimulate nerve fibers. In PN, periostin expression was reported to be correlated with itch and was independent of IL-31 expression, indicating an independent pathway of PN-related itch.^121^

Although scratching can temporarily relieve pruritus by activating fibers that block itch sensation in the spine, repeated scratching can cause mechanical disruption of the skin, leading to the aforementioned neural, immunologic, and epithelial changes that further exacerbate pruritus. In normal skin, scratching relieves itch by activating Piezo2 ion channels on Merkel cells, which simulate Ab fibers to reduce itch. In dry skin, the same signaling pathway can activate MRGPRA3-positive pruriceptors that exacerbate itch and drive the itch-scratch cycle.^122,123^ In addition to miswiring in the periphery, scratching also activates reward regions of the brain. Structural and functional changes have been observed in the brain’s motor and reward regions in patients with chronic pruritus, suggesting that the itch-scratch cycle may be perpetuated at the level of the central nervous system.^124^ An active area of exploration is the inflammatory cross talk between the periphery and the brain and the way in which inflammatory circuits within peripheral tissue can imprint durable alterations within the nervous system to drive pathologic sensation and neuroinflammation.

The physiologic mechanisms of itch can change with age, which may explain differences in pruritus across a patient’s lifetime. Merkel cells in the skin suppress mechanical itch in pruriceptive C neurons via cutaneous Piezo2 activation; these cells decrease in number with age.^122,123,125^ In addition, MRGPRA3-positive pruriceptors become more closely associated with Merkel cells in chronic itch models.^122,123^ An increase in alloknesis arising from a hypersensitive itch response, which is often seen in dry or aging skin, is correlated with the loss of Merkel cells with age.^122,123^ Similar observations in the oral mucosa may explain loss of ability to effectively chew and swallow with age.^126^ In addition, immunosenescence is a proinflammatory phenomenon that may, in some cases, be manifested as a type 2 imbalance.^127–130^ These findings may help explain the higher prevalence of PN in older adults and patients with other chronic pruritic conditions.^131,132^

Two mAbs, dupilumab and tralokinumab, have been approved for the treatment of AD in several countries,^133–135^ whereas a third mAb, nemolizumab, has been approved in Japan for treatment of itch associated with AD when other treatments are ineffective.^136^ In addition, on the basis of the results from 2 phase 3 trials, dupilumab was recently approved by the US Food and Drug Administration and the European Medicines Agency for treatment of PN.^133–135,137^ Dupilumab is a fully human VelocImmune-derived mAb that blocks IL-4Rα, preventing binding of IL-4 and IL-13.^51,138^ Multiple randomized placebo-controlled clinical trials of dupilumab (with or without concomitant topical corticosteroids) in adults, adolescents, children, and infants with moderate-to-severe AD have demonstrated that dupilumab significantly improves clinical signs and neuroimmune inflammation-driven symptoms (including itch), as well as other patient-reported outcomes.^20–26,139^ Tralokinumab and lebrikizumab both bind to IL-13 and have been shown to improve signs and symptoms of moderate-to-severe AD.^27–33^ Nemolizumab, an anti-IL-31 receptor 1 mAb, has demonstrated activity in AD and PN.^34,36,50,136^ JAK inhibitors (JAKIs), such as abrocitinib, baricitinib, and upadacitinib, are also approved and have demonstrated efficacy in AD.^37–41^

### Airway type 2 inflammation, hyperresponsiveness, and reduced expiratory volume in asthma

Asthma is a chronic respiratory disease characterized by airflow obstruction, increased mucus production, airway inflammation, and hyperresponsiveness. The pathophysiology of asthma is multifactorial, and multiple types of immune cells have been implicated in its development. Type 2 inflammation plays a critical role in asthma, as IL-4, IL-5, and IL-13 are key drivers of this atopic disease.^1,2,140–142^ These cytokines promote airway obstruction and tissue remodeling, including smooth muscle contraction, basement membrane thickening, eosinophilia, goblet cell hyperplasia, and mucus production.^2,140,142–144^ IL-4 and IL-13 induce production of eotaxin, which cooperates with IL-5 in promoting eosinophilia in the respiratory epithelium.^2,140^ IL-5 is a potent regulator of eosinophil growth, differentiation, migration and survival, and it plays a key role in asthma—particularly the eosinophilic phenotype.^1,142,145,146^ IL-31 may also contribute to the production of type 2 inflammatory factors and recruitment of immune cells in the lung.^147^

Similar to the bidirectional neuroimmune cross talk observed with the itch-scratch cycle in AD and PN, reciprocal neuroimmune interactions may perpetuate airway inflammation and sensory pathologies in asthma (Fig 3).^148^ Sensory neurons of the vagal ganglia densely innervate the conducting airways of the lung; those of the nodose ganglia can extend into the alveolar region.^149,150^ Very few DRG neurons innervate the lung; those that do project only to extrapulmonary large airways.^148–150^ Inhaled ovalbumin, a commonly used allergen in animal models of asthma, as well as type 2 inflammatory mediators such as IL-5, can directly activate calcium signaling in cultured nodose neurons.^75^ *In vivo* genetic ablation or chemical inactivation of voltage-gated sodium channel 1.8–expressing vagal nociceptor neurons has been shown to blunt allergen-induced increase in immune cells.^75^ However, in a separate study, *in vivo* genetic ablation of largely overlapping TRPV1-expressing vagal nociceptor neurons did not affect allergen-induced increases in immune cells.^151^ Thus, whether vagal sensory neurons are required for regulating allergen-induced T_H_2 cell airway immune responses remains unclear.

Neuropeptide release from sensory neurons may have a role in asthma. Levels of SP are increased in induced sputum and correlated with airway obstruction, and CGRP level is elevated in bronchoalveolar lavage fluid.^152,153^ Neuropeptide release by sensory neurons positive for TRPA1 or TRPV1, which are markers of nociceptors, may play a particularly important role in this setting.^154,155^ For example, in cultured vagal ganglia nociceptive neurons, IL-5 stimulates the release of neuropeptides, such as VIP, that stimulate CD4-positive T cells and ILC2s, which in turn release type 2 cytokines such as IL-5, thereby amplifying allergic inflammation. In contrast, silencing these neurons ameliorates airway inflammation.^75^ In addition, cholinergic neurons in the gut and lung release neuromedin U, which promotes ILC2 response.^59,60,156^ Recent studies have suggested that some lung mast cells may also express MRGPRX2, the expression of which is upregulated in patients with asthma and activated by neuropeptide hemokinin-1.^157–159^ How sensory neurons and autonomic neurons interact to regulate inflammation remains poorly understood, despite the availability of many therapies targeting adrenergic and cholinergic pathways in the airway. For example, CGRP and neuromedin U have divergent effects, demonstrating the complexity of the mechanisms by which neuropeptides regulate type 2 inflammation.^160,161^ The central nervous system has been shown to exert systemic anti-inflammatory effects via the vagus nerve by inhibiting splenic macrophages in the cholinergic anti-inflammatory pathway, and some evidence suggests that manipulation of the vagus nerve (with cholinergic agonists or vagal nerve stimulation) may modulate lung inflammation.^162^

Chronic respiratory inflammation can sensitize nerve fibers that innervate the airways, resulting in smooth muscle hypercontraction and airway constriction, ultimately resulting in airway hyperresponsiveness (AHR). AHR is correlated with the severity of asthma and is a cardinal feature associated with the degree of treatment necessary to control symptoms.^148^ In the human airway, mast cell numbers within the airway smooth muscle bundle are correlated with the severity of AHR.^57^ A murine model devoid of mast cells shows blunted AHR.^58^ Eosinophils also play a role in bronchoconstriction and are recruited by eotaxin to cluster at airway nerves; preclinical models suggest that IL-4 and IL-13 increase expression of eotaxin in parasympathetic neurons.^53^ Additional studies demonstrate a similar effect of both IL-4 and IL-13 in inducing smooth muscle hyperresponsiveness in explanted human lung tissue.^54–56^ IL-13 has also been shown to upregulate periostin in airway epithelium.^163^

Sympathetic and parasympathetic neurons (and their reciprocal interactions with sensory neurons and immune cells) contribute to the clinical manifestations of asthma.^46^ Sympathetic neurons release the neurotransmitter noradrenaline, which induces airway smooth muscle relaxation via β2-adrenergic receptors (β2Ars) on smooth muscle cells.^46^ β2AR agonists, which are often used as bronchodilators for the treatment of asthma, may influence immune cells. For example, β2AR agonists reduce IL-33–induced airway inflammation and suppress ILC2 proliferation.^46,62^ Immune cells also influence noradrenergic function: mast cell– derived transforming growth factor β triggers phosphorylation of β2AR on smooth muscle cells, which reduces the response to albuterol, a β2AR agonist.^164^ Finally, eosinophils increase sensory nerve responsiveness and density in the airway and release mediators that antagonize inhibitory M_2_ muscarinic receptors on parasympathetic nerves. The effects of eosinophils on both sensory and parasympathetic nerves potentiate bronchoconstriction.^165^

Rare sensory cell types in the lung epithelium also play important roles in sensing allergens and in asthma pathogenesis.^166,167^ Pulmonary neuroendocrine cells are activated by allergens to increase their production of neuropeptides, such as CGRP, and neurotransmitters, such as γ-aminobutyric acid. These cells are essential for amplifying allergen-induced responses, including activation of ILC2s, recruitment of T_H_2 immune cells, and goblet cell metaplasia.^63^ Similarly, tuft cells can detect allergens and release neurotransmitters (such as acetylcholine), alarmins (such as IL-25), and inflammatory mediators (such as leukotrienes) that can contribute to allergic inflammation in the airways.^168,169^ Tuft cells also promote respiratory reflexes such as sneezing and coughing.^167,169^

Six biologics have been approved for treatment of asthma: omalizumab, mepolizumab, reslizumab, benralizumab, tezepelumab, and dupilumab.^170,171^ Omalizumab is a humanized mAb directed against IgE.^170,171^ In multiple clinical trials, omalizumab significantly improved signs and symptoms of asthma; for example, it reduced exacerbation rates, improved lung function and symptoms of asthma, and reduced use of inhaled corticosteroids (vs oral corticosteroids).^171^ Mepolizumab, reslizumab, and benralizumab inhibit the IL-5 pathway (a key regulator of eosinophils) by targeting either IL-5 (mepolizumab and reslizumab) or the IL-5 receptor (benralizumab).^172^ All 3 were well tolerated and reduced asthma exacerbations in clinical trials in adults and adolescents with severe eosinophilic asthma that was otherwise inadequately controlled with standard therapies.^172^ Tezepelumab, which targets the epithelial cell–derived cytokine TSLP, reduced asthma exacerbation rates in a phase 3 trial,^173^ and in a long-term, randomized, placebo-controlled extension study, it continued to reduce exacerbation rates for up to 104 weeks.^174^ Dupilumab, which inhibits IL-4 and IL-13 signaling, reduced asthma exacerbations, improved lung function, and reduced oral corticosteroid use in clinical trials in patients with moderate-to-severe asthma—particularly in patients with elevated levels of eosinophils and/or fractional exhaled nitric oxide.^175–177^ Given the neuroimmune effects of eosinophils in asthma, it is possible that blockade of IL-4, IL-5, IL-13, and TSLP may alter these effects, thereby addressing some of the underlying pathophysiologic mechanisms of asthma.

These therapeutics reveal important differences in the tissue-specific effects of type 2 cytokines. Although anti–IL-5 therapies and tezepelumab have been successful and approved for use in asthma, they have not been successful in AD. Therapies specifically targeting IL-13 alone have advanced in AD but have failed in asthma. However, dual blockade of IL-4 and IL-13 has been successful in both diseases. Notably, although the IL-5 receptor has been reported in the nodose ganglia of the lung,^75^ the DRGs for the skin lack this receptor.^18^

### Loss of smell in CRSwNP

CRSwNP is a chronic inflammatory disease of the nasal mucosa and paranasal sinuses. Clinical manifestations include nasal congestion, rhinorrhea/postnasal drip, facial pain or headache, and impaired sense of smell. Notably, pruritus often accompanies CRSwNP, and increased levels of periostin have been found in the nasal mucosa of patients with CRSwNP.^121^ Levels of IL-31 are also increased in patients with allergic rhinitis (AR); in this setting, IL-31 is induced by IL-4 and promotes type 2 inflammation.^50^ Olfactory dysfunction in CRSwNP is common and negatively affects quality of life.^178,179^ The mechanisms underlying olfactory dysfunction in CRSwNP are multifaceted, involving changes to the olfactory mucosa following (and related to) the type 2 inflammatory response, disruption of synaptic transmission resulting from neuroepithelial edema, and decreased airflow in the olfactory cleft (Fig 3).^180,181^ Nasal obstruction may contribute to olfactory dysfunction, but it is unlikely to be the primary driver because the sense of smell does not always return after polypectomy.^182,183^ However, the extent of inflammation in the neuroepithelium is correlated with the severity of olfactory dysfunction.^184^

CRSwNP has a prominent type 2 inflammatory signature.^185^ Studies of mucosal tissue in patients with nasal polyps (and other studies using a mouse model of chronic rhinosinusitis) have demonstrated elevated numbers and increased activation of T cells; infiltration of nasal polyps by eosinophils, basophils, and mast cells; and type 2 polarization with elevated levels of IL-4, IL-5, IL-13, and IgE.^65,185–187^ Release of inflammatory mediators triggers hypersecretion of olfactory mucus, resulting in changes in ion concentration that can affect the activity of olfactory neurons.^187^ In a mouse model of chronic rhinosinusitis, chronic type 2 inflammation reduced the number of immature neurons, suggesting that chronic inflammation may inhibit olfactory sensory neurogenesis, rendering the olfactory mucosa less able to recover from injury.^65^ In another mouse study, IL-4Rα was expressed in olfactory sensory neurons; however, although both IL-4 and IL-13 increased calcium uptake (indicating activation) in these neurons, mice developed anosmia following intranasal administration of IL-4, but not IL-13.^64^

Transient receptor potential channels such as TRPA1, TRPV1, transient receptor potential vanilloid 4 (TRPV4), transient receptor potential cation channel subfamily M member 4 (TRPM4), and transient receptor potential cation channel subfamily M member 8 (TRPM8) are expressed in the upper airway in epithelial cells, sensory neurons, T cells, and mast cells.^66^ These channels respond to physical stimuli (eg, changes in temperature) and chemical stimuli, and they are associated with type 2 inflammatory processes in the upper airway.^66^ For example, desensitization or ablation of TRPV1-positive sensory fibers in mice reduced allergy-associated and chemically induced sneezing, which were mediated by neuropeptides such as neuromedin B.^67^ In another mouse model of AR, knockout or inhibition of TRPV1 suppressed T_H_2/T_H_17 cytokine production, eosinophil infiltration, and IgE in nasal mucosa.^68^ Similarly, patients with AR had higher numbers of TRPV1-positive cells in the nasal mucosa than healthy controls did.^68^

Recent studies suggest that biologics may improve the olfactory changes associated with CRSwNP. Currently, 3 biologics are approved for CRSwNP: omalizumab, mepolizumab, and dupilumab. Omalizumab improved clinical and endoscopic aspects of CRSwNP, including sense of smell.^188,189^ Mepolizumab improved nasal obstruction in a phase 3 trial, with modest improvement in sense of smell.^190^ Dupilumab reduced polyp size, reduced symptom severity, improved sense of smell and quality of life, and was well tolerated in 3 phase 3 trials of adults with CRSwNP.^190–193^ Notably, improvement in sense of smell was rapid in patients treated with dupilumab.^191–193^ IL-4Rα is present on olfactory sensory neurons^64^; however, further research is needed to elucidate the relationship between this receptor and the neuroimmune mechanisms underlying such swift restoration of olfaction.

### Dysphagia in EoE

EoE is a chronic inflammatory disease of the esophagus. Primary symptoms include dysphagia and food impaction in adults and failure to thrive, feeding difficulties, and vomiting in infants and children.^194,195^ These symptoms are consistent with dysregulated neural control of the esophagus and inflammation-driven tissue remodeling.^69,70,196^ EoE is characterized by a type 2 inflammatory response (Fig 3). Type 2 inflammation–derived nociceptor sensitization of the esophagus leads to pain, motor dysfunction and eventually tissue remodeling.^69,70^ Following exposure to food-related antigens, esophageal epithelia secrete TSLP and IL-33, causing T_H_2 cells to release IL-4, IL-5, and IL-13. The presence of IL-4 and IL-13 induce dilated intracellular spaces and basal cell hyperplasia in the esophageal epithelia, whereas IL-5 and eotaxin-3 promote infiltration by eosinophils and possibly by mast cells and basophils.^70,195,197^ However, some experts suggest that dysphagia is mostly related to tissue remodeling.^198–200^

Increased infiltration of eosinophils and mast cells in esophageal tissue may increase vagal sensory neuronal responsiveness to acid, promoting barrier dysfunction and impaired epithelial permeability.^71,72^ Increased epithelial permeability may enhance the ability of luminal acid to stimulate action potential discharge in nociceptive afferent terminals. Type 2 cytokines can also cause hypercontractility of gastrointestinal smooth muscle cells via signal transducer and activator of transcription 6 (STAT6) or mitogen-activated protein kinase (MAPK) signaling pathways, which may contribute to dysphagia.^71^

TRPV1 and mast cells may modulate pain in EoE. In a study in patients with EoE with pain, pain was positively associated with molecular expression of TRPV1, carboxypeptidase A3, and hematopoietic prostaglandin D synthase but not eosinophilia; mast cells colocalized with neurons, which is suggestive of mast cell specificity.^73^ Neuropeptides such as SP and VIP promote mast cell degranulation, the production of type 2 cytokines and chemokines, and a resultant immune cascade.^7^ Because TRPV1-positive sensory neurons are prominent within the vagus nerve and have itch-specific transcriptional identity at the single-cell level,^201^ symptoms of discomfort and irritation associated with EoE may be defined by molecular and cellular circuits that resemble itch mechanisms rather than traditional pain-associated pathways.

In clinical studies, agents targeting the IL-5 pathway (mepolizumab and reslizumab) have been shown to reduce eosinophil numbers in esophageal tissue but had minimal impact on symptoms.^202–204^ Similarly, lirentelimab, which targets sialic acid–binding immunoglobulin-like lectin 8 (Siglec-8), reduced eosinophil counts but failed to improve symptoms in patients with EoE.^205^ Agents targeting IL-13 have produced positive results; they include the anti–IL-13 mAbs QAX576 and RPC4046^206,207^ and dupilumab, which inhibits both IL-4 and IL-13 signaling.^208,209^ Dupilumab is the first US Food and Drug Administration–approved drug for the treatment of EoE. In clinical trials in patients with EoE, dupilumab reduced peak intraepithelial eosinophil counts in esophageal tissues and improved clinical, histologic, and endoscopic scores.^208,209^

Thus, the success of therapeutics targeting the type 2 inflammation spectrum is highly dependent on tissue-specific mechanisms. Future studies will be required to determine how epithelial, stromal, and neural pathways, influenced by type 2 cytokines, imprint unique responses within specific tissues.

## DISCUSSION

Multiple links connect neuroimmune cross talk, inflammatory pathways, and sensory pathologies across type 2 inflammatory diseases. Type 2 inflammation promotes alterations in neural and immune effectors and their interactions, leading to both sensory and autonomic dysfunction in AD, PN, asthma, CRSwNP, and EoE. Reciprocal neuroimmune interactions may perpetuate inflammation and sensory pathology in these diseases. For example, in AD, strong evidence supports a role for type 2 inflammation in a positive feedback loop of sustained neural and immunologic changes that drive sensory and behavioral pathology (ie, the itch-scratch cycle), which may contribute to the chronic nature of AD. In contrast, PN exemplifies how a potentially neurogenic process can also be linked to type 2 inflammation and drive inflammatory and fibrotic pathology in the skin. Neuroimmune changes have been well characterized in AD and asthma, but such changes are less well characterized in PN, CRSwNP, and EoE. Interestingly, studies in multiple type 2 inflammatory diseases have demonstrated that mast cells colocalize with afferent nerve terminals in epithelial tissues and sensitize nociceptive C-fibers.^7,8,48,57,65,69,73,164,186^ As such, these cells may constitute a shared element in type 2 inflammatory diseases at different epithelial surfaces and neuroimmune manifestations. Further research is needed to better understand whether similar feedback loops also occur in other organs susceptible to type 2 inflammatory manifestations.

**FIG 4.**
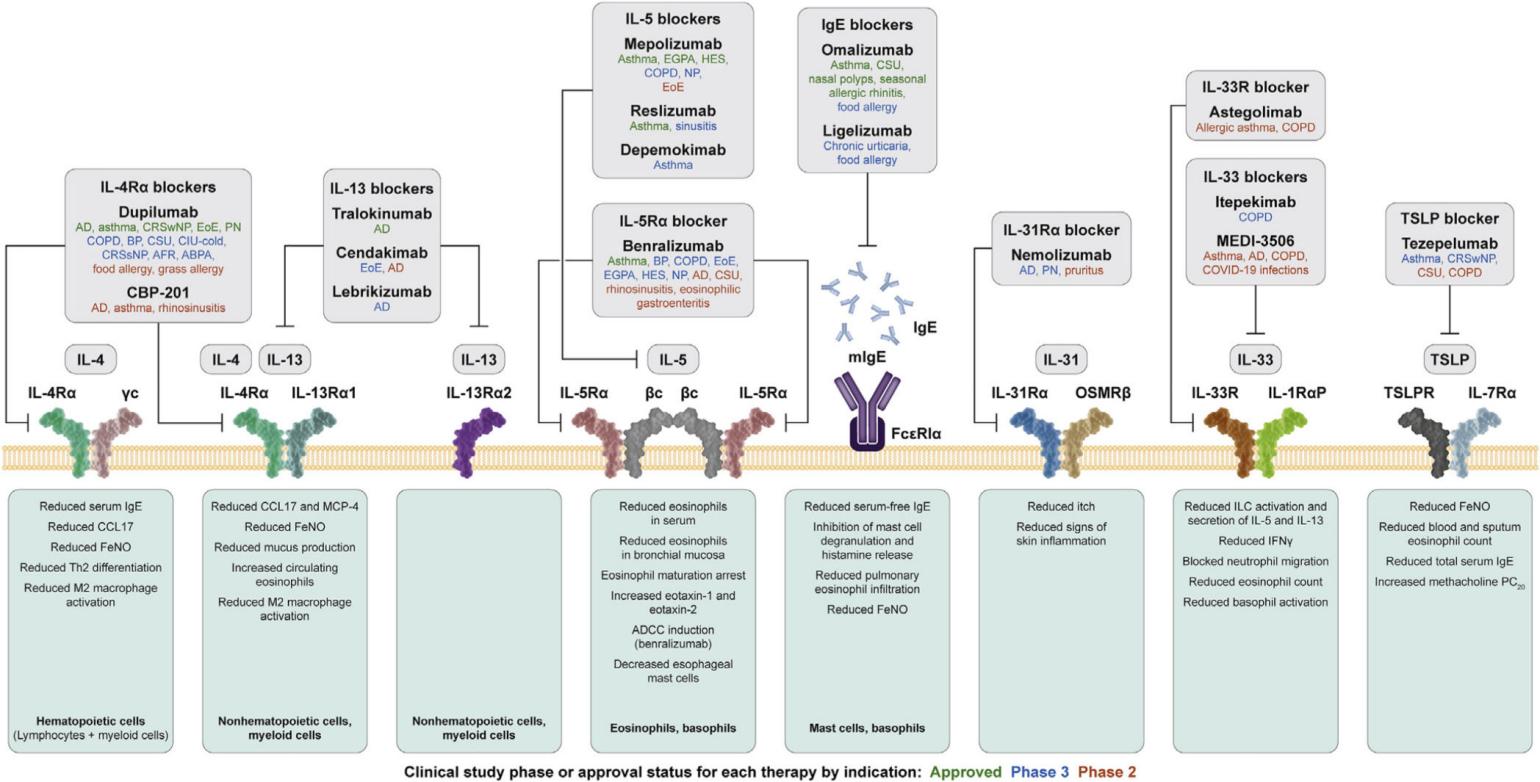
Biologics that inhibit type 2 inflammation. *βc*, β-chain; *γc*, γ-chain; *ABPA*, allergic bronchopulmonary aspergillosis; *ADCC*, antibody-dependent cellular cytotoxicity; *AFR*, allergic fungal rhinosinusitis; *BP*, bullous pemphigoid; *CCL17*, C-C motif chemokine ligand 17; *CIU*, chronic idiopathic urticaria; *COPD*, chronic obstructive pulmonary disorder; *CRSsNP*, chronic rhinosinusitis without nasal polyps; *CSU*, chronic spontaneous urticaria; *EGPA*, eosinophilic granulomatosis with polyangiitis; *F*_*ENO*_, fractional exhaled nitric oxide; *HES*, hypereosinophilic syndrome; *IL-1RαP*, IL-1 receptor-α P; *IL-5Rα*, IL-5 receptor-α; *IL-13Rα1/2*, IL-13 receptor-α1/2; *IL-31RA*, IL-31 receptor A; *IL-33R*, IL-33 receptor; *ILC*, innate lymphoid cell; *MCP-4*, monocyte chemoattractant protein-4; *mIgE*, membrane IgE; *NP*, nasal polyps; *OSMRβ*, oncostatin-M specific receptor subunit beta; *PC*_*20*_, provocative concentration causing a 20% drop in forced expiratory volume in 1 second from baseline; *TSLPR*, thymic stromal lymphopoietin receptor. Figure adapted with permission from Haddad EB et al.^220^

Traditional systemic treatments for type 2 inflammatory diseases include broad-acting immunosuppressants (such as corticosteroids, cyclosporine, methotrexate, azathioprine, and mycophenolate mofetil); however, their use is limited by safety concerns and a heightened risk of infection.^210–214^ Further, these broadly immunosuppressive agents do not target neural pathways and do not demonstrate the efficacy seen with more targeted therapeutics. Recent approaches to selective suppression of type 2 inflammation include biologics that target cytokines or their receptors, including dupilumab, lebrikizumab, nemolizumab, tralokinumab, omalizumab, mepolizumab, reslizumab, benralizumab, tezepelumab, and vixarelimab,^17,20–36,52,133,134,137,139,170–177,202–209,215–217^ as well as JAKIs that target the intracellular JAK/STAT signaling pathways, including abrocitinib, baricitinib, and upadacitinib.^37–41^ In AD, improvement in pruritus observed with the IL-13 inhibitors tralokinumab^27–30^ and lebrikizumab^31–34^ suggests potential modulation of underlying neuroimmune pathology by IL-13 inhibition. In AD and PN, improvements in pruritus with dupilumab^20–26,133,134,137,139,215,217^ suggest that inhibiting signaling of both IL-4 and IL-13 through IL-4Rα blockade could control multiple manifestations of aberrant neuroimmune activity in the broader context of type 2 inflammation, including IgE-mediated acute itch flares.^13,218,219^ JAKI treatment has also been shown to improve pruritus in patients with AD, demonstrating the importance of the JAK/STAT pathways in modulating type 2 inflammatory effects on pruritus.^37–41^ In asthma, the complex interactions among immune cells, type 2 inflammatory cytokines, and sensory neurons indicate that biologics suppressing type 2 inflammation could have neuroimmune effects that improve airway function.^170–177^ In patients with CRSwNP, improvements in the sense of smell following treatment with biologics (omalizumab, mepolizumab, and dupilumab)^188–193^ suggest that biologic therapy may have direct neuroimmune effects on olfactory sensory neurons that go beyond simple reductions in nasal obstruction and may offer improvements that are not often achieved with surgery. Lastly, EoE is a type 2 inflammatory disease, and key symptoms of dysphagia and pain suggest a neurologic component. In EoE, persistence of symptoms despite histologic remission suggests that changes to sensory neurologic pathways may also persist and may be epigenetically mediated. The fact that symptom improvement in patients with EoE has been observed in clinical trials of dupilumab,^208,209^ but not with other biologics (such as IL-5 and sialic acid–binding immunoglobulin-like lectin 8 [Siglec-8] inhibitors),^203–205^ provides insight into neuroimmune factors that are particularly relevant to EoE symptoms. Additional biologics have been developed, or are under investigation, for the treatment of sensory pathologies in atopic diseases. These biologics and their targets are shown in Fig 4.^220^

Further research is needed to better understand the neuroimmune mechanisms underlying chronic, sustained inflammation and its related sensory pathologies in diseases associated with type 2 inflammation, which will undoubtedly inform the development of new treatment strategies and treatment selection for patients with these diseases.

## Figures and Tables

**FIG 1.**
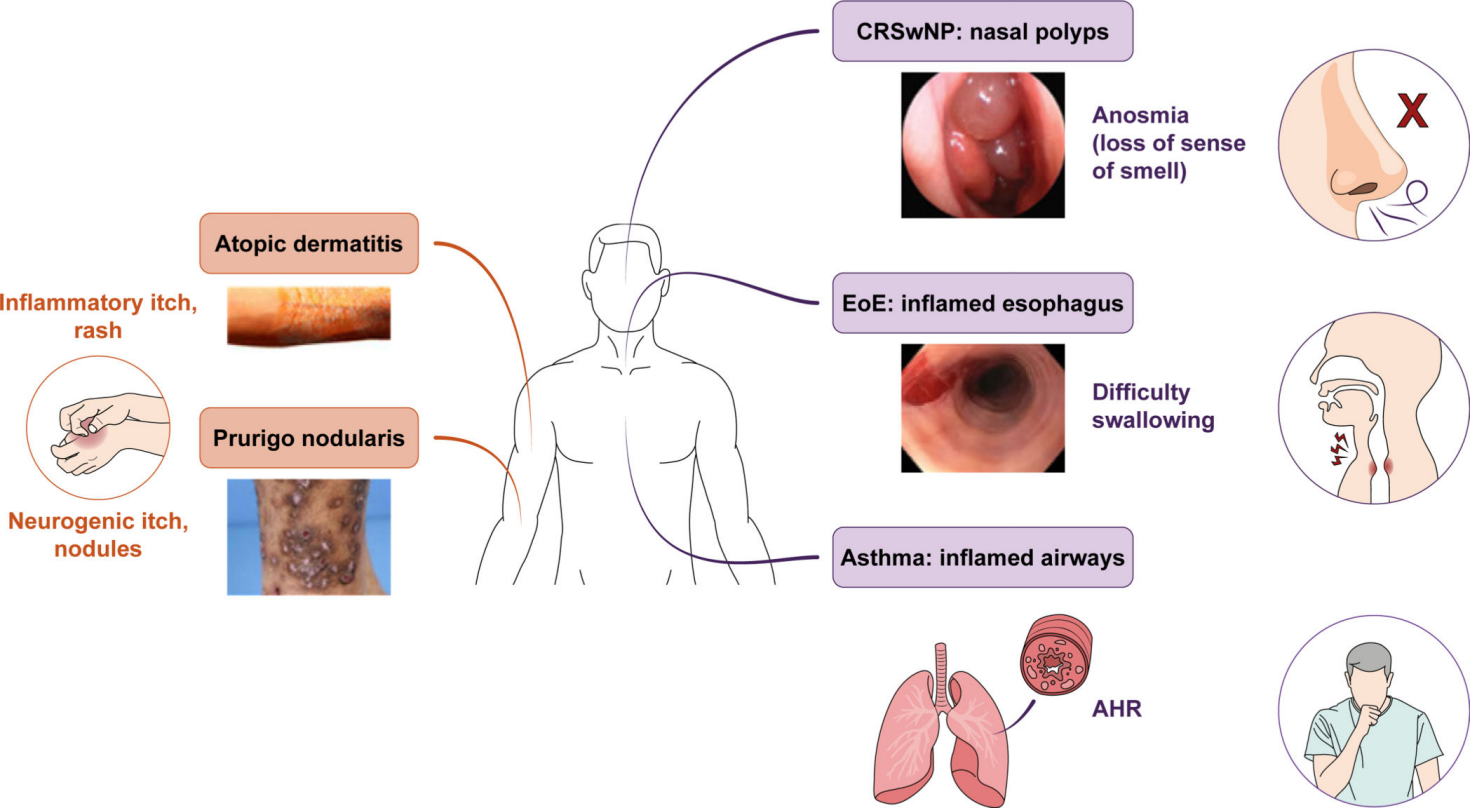
Sensory pathologies in atopic diseases.

**FIG 2.**
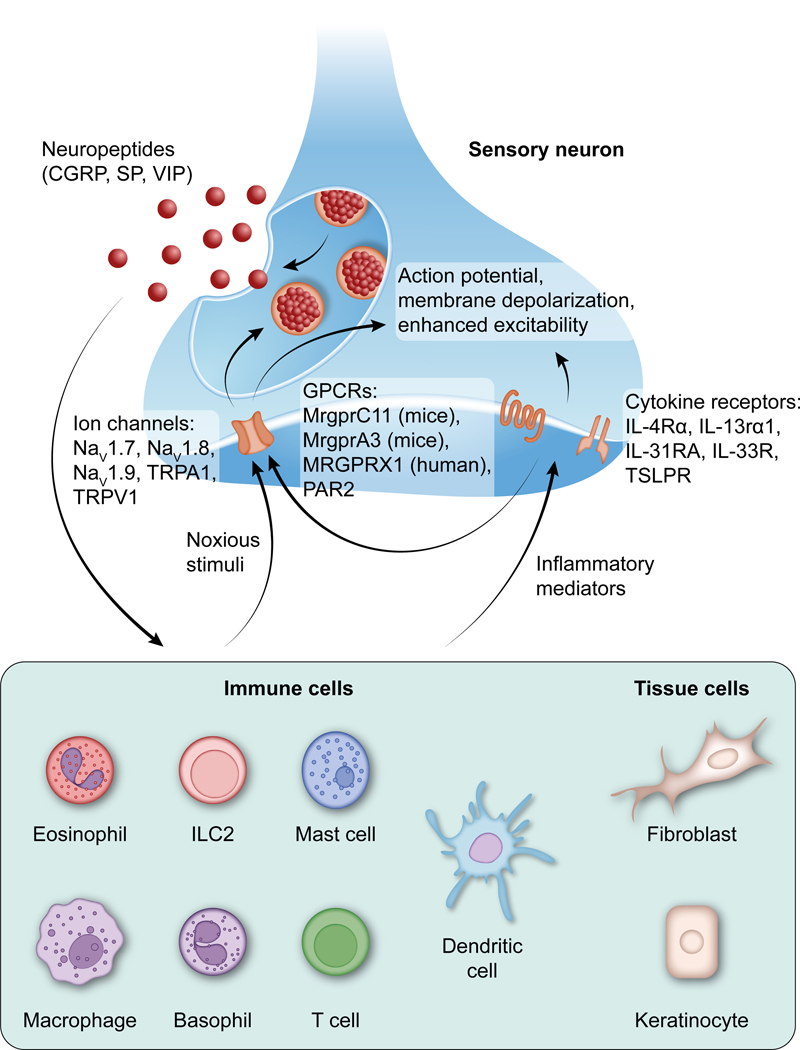
Type 2 inflammation and sensory neurons: bidirectional cross talk. Stimulated neurons produce neuropeptides that can modulate the activity of immune cells, including the production of inflammatory mediators that can directly affect sensory neurons. *IL-13Rα1*, IL-13 receptor-α 1; *IL-31RA*, IL-31 receptor A; *IL-33R*, IL-33 receptor; *MrgprCr11*, *Mas*-related family of G protein-coupled receptor 11; *MRGPRX1*, *Mas*-related family of G protein-coupled receptor X1; *Na*_*v*_, voltage-gated sodium channel; *NMU*, neuromedin U; *PAR2*, protease activated receptor 2; *TSLPR*, thymic stromal lymphopoietin receptor.

**TABLE I.** Neuroimmune interactions affecting symptoms in AD, PN, asthma, CRSwNP, and EoE

Condition	Symptoms	Neuroimmune interactions	Type 2 cytokine profile
AD	Pruritus	● Colocalization of neurons and immune cells^7,8^ ● Type 2 cytokines and OSM promote pruritogen sensitization^9^ ● Neuropeptide release from sensory neurons induces proinflammatory cytokines and activates immune cells (eg, mast cells, basophils)^7,8,10–13^ ● Scratching causes release of alarmins (eg, IL-33), which can act directly on sensory neurons^9,11,14–17^ ● Some sensory neurons express receptors for IL-4, IL-13, IL-31, TRPA1, TRPV1^18,19^ ● Reducing type 2 inflammation reduces itch^20–36^ ● Inhibition of IL-4–mediated JAK/STAT signaling reduces itch^37–41^	Prominent role: IL-4, IL-13, IL-31 Limited role: IL-15, periostin
PN	Pruritus	● Colocalization of neurons and immune cells^7,8^ ● Sensory neuron dysfunction and increased density of SP- and CGRP-immunoreactive nerve fibers^42–47^ ● Neuropeptide release from sensory neurons induces proinflammatory cytokines and activates immune cells (eg, mast cells, basophils)^7,8,11,43,45–48^ ● Increased numbers of IL-31–positive cells and OSM-positive cells are correlated with itch intensity^49,50^ ● Reducing type 2 inflammation also reduces itch^36,51,52^	Prominent role: IL-4, IL-13, IL-31, periostin
Asthma	AHR	● Colocalization of neurons and immune cells^53^ ● Type 2 cytokines can stimulate neuropeptide release from sensory neurons (IL-5) and induce AHR (IL-4 and IL-13)^54–56^ ● Chronic inflammation sensitizes nerve fibers in airways, promoting AHR^53–58^ ● Airway neurons can enhance allergen-induced immune cell response, including ILC2 response via NMU^59,60^ ● Eosinophils increase sensory nerve density and responsiveness, promoting bronchoconstriction^61^ ● β2AR agonists mediate sympathetic nerve activity, IL-33–induced inflammation, and ILC2 proliferation^45,62^ ● Pulmonary neuroendocrine cells enhance allergen-induced response by recruiting T_H_2 immune cells and activating ILC2s^63^	Prominent role: IL-4, IL-5, IL-13, IgE
CRSwNP	Anosmia	● Olfactory sensory neurons express IL-4Rα^64^ ● Chronic type 2 inflammation inhibits olfactory neuron function and neurogenesis^65^ ● TRP channels on sensory neurons and immune cells in the upper airway are associated with type 2 inflammatory processes^66–68^	Prominent role: IL-4, IL-5, IL-13, IgE, periostin
EoE	Dysphagia	● Type 2 inflammation sensitizes nociceptors, causing pain, motor dysfunction, and tissue remodeling^69,70^ ● Type 2 cytokines can induce hypercontractility of smooth muscle cells via STAT6 or MAPK^71^ ● Infiltrating immune cells can increase vagal sensory neuron responsiveness to acid^71,72^ ● Pain severity is linked to TRPV1 and mast cells^73^	Prominent role: IL-4, IL-13 Limited role: IL-5, Siglec-8

*MAPK*, Mitogen-activated protein kinase; *NMU*, neuromedin U; *Siglec-8*, sialic acid–binding immunoglobulin-like lectin 8; *TRP*, transient receptor potential.

## Abbreviations used

AD: Atopic dermatitis
AHR: Airway hyperresponsiveness
AR: Allergic rhinitis
β2AR: β2-adrenergic receptor
CGRP: Calcitonin gene–related peptide
CRSwNP: Chronic rhinosinusitis with nasal polyps
DRG: Dorsal root ganglion
EoE: Eosinophilic esophagitis
IL-4Rα: IL-4 receptor alpha
IL-31RA: IL-31 receptor A
ILC2: Group 2 innate lymphoid cell
JAK: Janus kinase
JAKI: JAK inhibitor
MrgprA: *Mas*-related family of G-protein-coupled receptor
MrgprA3: *Mas*-related family of G-protein-coupled receptor 3
OSM: Oncostatin M
PN: Prurigo nodularis
SP: Substance P
STAT: Signal transducer and activator of transcription
TRPA1: Transient receptor potential ankyrin 1
TRPV1: Transient receptor potential vanilloid 1
TSLP: Thymic stromal lymphopoietin
VIP: Vasoactive intestinal peptide
